# Supplementary material for: Transcriptome Analysis Reveals Vimentin-Induced Disruption of Cell–Cell Associations Augments Breast Cancer Cell Migration
Source: Cells. 2022 Dec 13;11(24):4035. doi: 10.3390/cells11244035 (PMC9776984; doi:10.3390/cells11244035)
Supplement: Supplementary file 1 [file cells-11-04035-s001.zip › DEGs and enrichment analysis.zip!/DEGs and enrichment analysis/WT_VIMvsVector GO enrichment/DOWN/WT_VIMvsVector_down.GOmf_DAG.pdf]

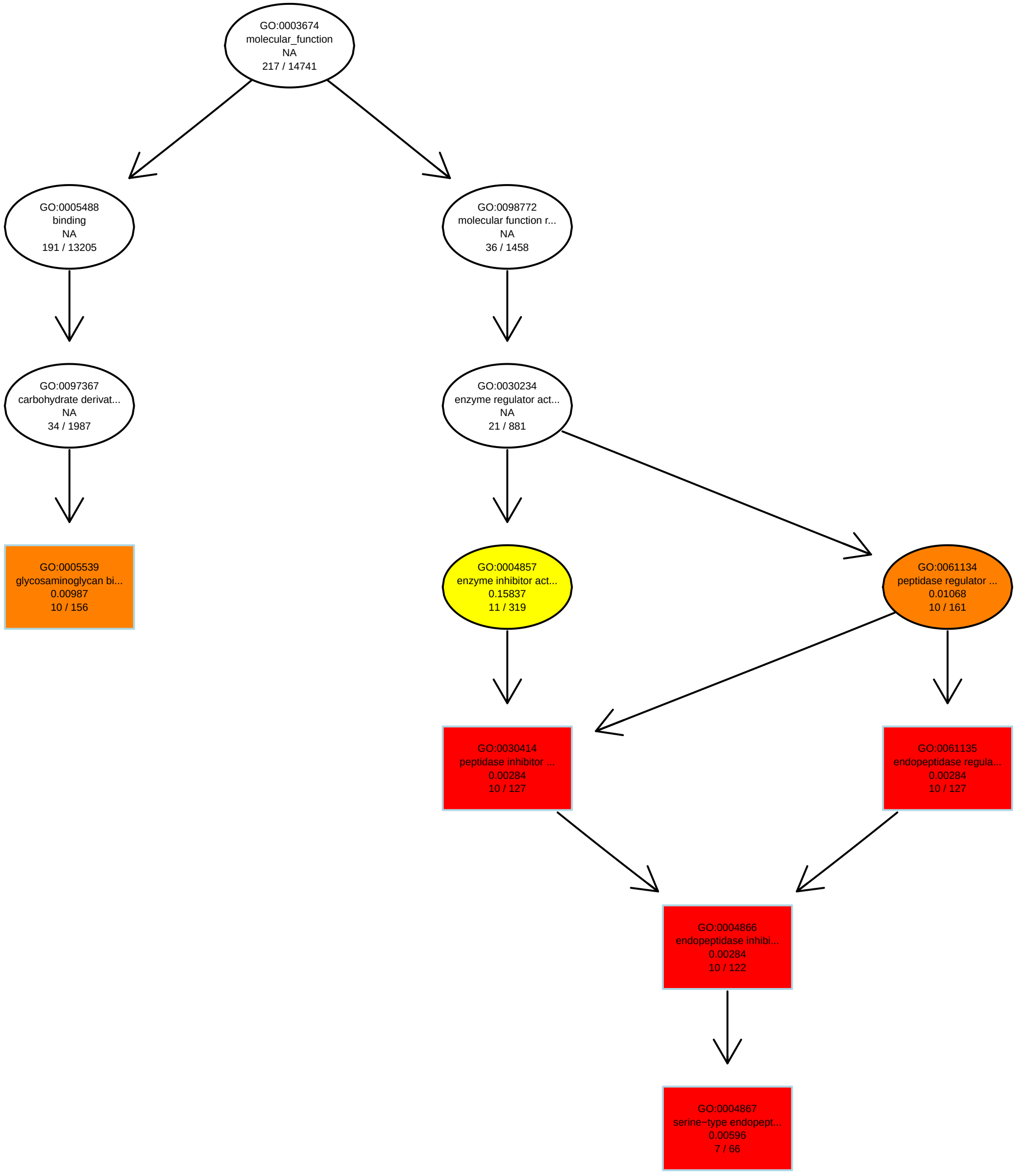
GO:0003674
molecular_function
NA
217 / 14741
GO:0005488
binding
NA
191 / 13205
GO:0098772
molecular function r...
NA
36 / 1458
GO:0097367
carbohydrate derivat...
NA
34 / 1987
GO:0030234
enzyme regulator act...
NA
21 / 881
GO:0005539
glycosaminoglycan bi...
0.00987
10 / 156
GO:0004857
enzyme inhibitor act...
0.15837
11 / 319
GO:0061134
peptidase regulator ...
0.01068
10 / 161
GO:0030414
peptidase inhibitor ...
0.00284
10 / 127
GO:0061135
endopeptidase regula...
0.00284
10 / 127
GO:0004866
endopeptidase inhibi...
0.00284
10 / 122
GO:0004867
serine−type endopept...
0.00596
7 / 66
